# Overexpression of HSPA2 is correlated with poor prognosis in esophageal squamous cell carcinoma

**DOI:** 10.1186/1477-7819-11-141

**Published:** 2013-06-18

**Authors:** Hang Zhang, Wei Chen, Chao-Jun Duan, Chun-Fang Zhang

**Affiliations:** 1 Department of Cardiothoracic Surgery, Xiangya Hospital, Central South University, Xiangya Road 87, Changsha, Hunan Province 410008, People’s Republic of China; 2 Medical Science Institute, Xiangya Hospital, Central South University, Xiangya Road 87, Changsha, Hunan Province 410008, People’s Republic of China

**Keywords:** HSPA2, Esophageal squamous cell carcinoma, Clinicopathology, Metastasis, Prognosis

## Abstract

**Background:**

Heat shock-related 70 kDa protein 2 (HSPA2) has been identified as a potential cancer-promoting protein expressed at abnormal levels in a subset of cancers. However, its important role in esophageal squamous cell carcinoma (ESCC) is hardly known by people. The purpose of this study is to assess HSPA2 expression and to explore its role in ESCC.

**Methods:**

Thirty ESCC samples, paired adjacent non-cancerous tissues and normal esophageal tissues, were collected for HSPA2 detection by quantitative RT-PCR (qRT-PCR) and western blotting. Additionally, the expression of HSPA2 in ESCC and adjacent non-cancerous tissues from 120 patients was analyzed by immunohistochemistry, and correlated with clinicopathological parameters and patients’ outcome.

**Results:**

HSPA2 mRNA and protein were overexpressed in ESCC tissues. Overexpression of HSPA2 was significantly associated with primary tumor, TNM stage, lymph node metastases and recurrence, respectively (all, *P* <0.05). Kaplan-Meier curves showed that elevated HSPA2 expression was associated with shorter disease-free survival and overall survival in ESCC patients. Cox multivariate regression analysis revealed that overexpression of HSPA2 was an independent prognostic factor in disease-free survival and overall survival for ESCC patients (hazard ratio was 2.115 and 2.210, respectively, *P* <0.05).

**Conclusions:**

Our findings demonstrate that overexpression of HSPA2 may contribute to the malignant progression of ESCC and present a novel prognostic indicator for ESCC patients.

## Background

Esophageal cancer is the seventh leading cause of cancer deaths worldwide, while its highest incidence and mortality rates are found in Asia. For example, in China, the majority of esophageal cancer diagnoses are esophageal squamous cell carcinoma (ESCC), which is ranked as the eighth leading cause of death nationwide, mostly in northern China where the incidence rate can be as high as 800 cases per 100,000 people [1]. The incidence and mortality rates of ESCC have decreased over the last few decades in China. On the contrary, it is continuing its march as the fastest growing malignancy in the Western world [2]. ESCC patients have a high mortality rate and poor prognosis because of the high prevalence of invasion and metastasis. Therefore, it is important to identify and characterize the tumor-specific molecular markers involved in early stages of ESCC that may contribute to ESCC carcinogenesis for improving the survival of this disease.

Heat shock-related 70 kDa protein 2 (HSPA2, also known as HSP70-2) is a member of the HSP70 family of heat-shock proteins, mapped to 14q24.1 in one study [3] and to 14q22 in another [4]. HSPA2 was originally found specifically in primary spermatocytes and spermatids, described as a testis-specific protein that played an important role in spermatogenesis [5,6]. Aberrant expression of HSPA2 in testes induced primary spermatocytes to arrest in meiosis I and undergo apoptosis, which was leading to male infertility [6]. Now HSPA2 has attracted increased interest due to its possible involvement in carcinogenesis of non-testicular tissues. HSPA2 has been identified as a potential cancer-promoting protein expressed at abnormal levels in a subset of human cancers, such as breast cancer [7], cervical cancer [8], bladder urothelial cancer [9], nasopharyngeal carcinoma [10] and malignant tumors [11]. Some level of the *HSPA2* gene activity was also observed in cell lines derived from several human cancers [12-14], while silencing of the *HSPA2* gene in cancer cells led to growth arrest and decrease in tumorigenic potential [8,9,12,15]. A HSPA2 mutation was recognized by cytotoxic T lymphocyte (CTL) on a human renal cell carcinoma [16]. Furthermore, polymorphism in the *HSPA2* gene is associated with an increase in the risk of developing type 1 diabetes [17], non-Hodgkin's lymphoma [18], lung cancer [19], systemic lupus erythematosus (SLE) [20], rheumatoid arthritis [21] and inflammatory bowel diseases [22]. Overexpression of HSPA2 is correlated with increased cell proliferation, poor differentiation and lymph node metastases in human breast cancer, cervical cancer and bladder urothelial cancer [7-9]. The highest level of HSPA2 was also detected in cells of the basal layers of the skin, esophagus and bronchus epithelia. However, whether there is HSPA2 expression in ESCC or not, has not been reported in China and abroad so far.

Thus, in this study we used quantitative RT-PCR (qRT-PCR), western blotting and immunohistochemistry to evaluate HSPA2 expression and its correlation with the survival of ESCC patients. Consistent with these studies, we suggested that elevated HSPA2 expression was associated with poor survival of ESCC patients. Our findings provide insights that could lead to better diagnosis, prognosis and therapeutic opportunities for ESCC patients.

## Methods

### Patients and tissue specimens

Primary ESCC samples, paired adjacent non**-**cancerous tissues (from 2 to 3 cm away from the tumor margin) and normal tissues (greater than 7 cm away from the tumor margin) were obtained from 30 patients (20 males and 10 females, median age 52.73 years, range 35 to 69 years). The patients underwent esophagus resection between January 2010 and June 2011 at Xiangya Hospital, Central South University, Changsha, China. All patients were validated by two pathologists. Before surgery, informed consent was obtained from all patients, whose specimens were handled and made anonymous according to the ethical and legal standards. The study was approved by the Research Ethics Committee of Central South University.

For immunohistochemical assays, 120 pairs of paraffin-embedded ESCC samples, adjacent non-cancerous tissues and normal tissues were obtained from patients who underwent curative resection between January 2004 and June 2007 at Xiangya Hospital. All patients had no history of previous malignancies and no history of radiotherapy or chemotherapy. Recurrence and metastasis were diagnosed by imaging evaluation, clinical examination, operation and postoperative pathological examination. In addition, the patients involved in this study did not have any other diseases which could cause infertility. The main clinical and pathological variables of the patients are recorded in Table 1. The follow-up time was 5 years for 120 patients, ranging from 5 months to 60 months.

**Table 1:** Correlation between HSPA2 expression and clinicopathologic features of ESCC patients (n = 120)

**Parameters**	**Cases**	**HSPA2 expression (n)**		**χ**^**2**^	***P *value**
		**Positive**	**Negative**		
Tissues					
Cancer	120	90	30	21.343	0.000
Adjacent	120	55	65		
Age (years)					
≤60	81	64	17	2.140	0.144
>60	39	26	13		
Gender					
Male	90	69	21	0.533	0.465
Female	30	21	9		
Drinking					
Yes	75	59	16	1.434	0.231
No	45	31	14		
Tumor site					
Upper	35	26	9	0.017	0.992
Middle	40	30	10		
Lower	45	34	11		
Grade					
G1	63	49	14	0.672	0.715
G2	30	21	9		
G3 + G4	27	20	7		
Primary tumor					
T1	30	18	12	9.340	0.025^a^
T2	31	21	10		
T3	42	35	7		
T4	17	16	1		
TNM stage					
I	28	14	14	14.991	0.001^a^
II	49	37	12		
III	43	39	4		
Lymph node metastases					
N0	76	51	25	6.890	0.009^a^
N+	44	39	5		
Recurrence^b^					
Yes	85	70	15	8.497	0.004^a^
No	32	18	14		

^a^*P* values considered statistically significant at <0.05; ^b^three patients lost to follow-up because of telephone number changes or home moving. ESCC, esophageal squamous cell carcinoma; G, grade; N, lymph node metastases; T, primary tumor. Drinking alcohol is defined as average amount of drinking alcohol is 50 grams or more per day, and period of drinking continues to 1 year or more. Drinking alcohol parameter: amount of drinking/day **×** years of drinking.

### qRT-PCR

Total RNA was isolated by TRIzol extraction liquid (Invitrogen, Carlsbad, CA, USA). Total RNA (2 μg) was reverse transcribed by cDNA Reverse Transcription Kits (Invitrogen) according to the manufacturer’s instructions. Primers were designed and synthesized by Sangon Biological Engineering Technology and Services (Shanghai, China). The primers for HSPA2 and β-actin were designed as follows: HSPA2 primer (196 bp), forward 5’-AAAACAAAATCACCATCACCAAT-3’, reverse 5’-CTAATCTTGCCCCTCAGTTTCTC-3’; β-actin primer (205 bp), forward 5′-TGACGTGGACATCCGCAAAG-3′, reverse 5′-CTGGAAGGTGGACAGCGAGG-3′. qRT-PCR was performed with SYBR Green PCR Master Mix according to the manufacturer’s instructions by using the CFX96 sequence detection system (Bio-Rad, Hercules, CA, USA) and accompanying analytical software. The reaction was first denatured at 95°C for 10 minutes, then 40 cycles at 95°C for 10 seconds, 60°C for 20 seconds and followed by 72°C for 10 seconds.

### Western blotting

HSPA2 protein was extracted using a Total Protein Extraction Kit (Beyotime, Haimen, Jiangsu, China). Briefly, all proteins were resolved on 10% SDS-PAGE in running buffer and transferred to polyvinylidene difluoride membranes (Millipore, Billerica, MA, USA). After blocking with 5% milk for 2 hours, the membranes were incubated with primary antibodies against HSPA2 (Santa Cruz Biotechnology, Dallas, TX, USA) and glyceraldehyde 3-phosphate dehydrogenase (GAPDH, Beyotime) at room temperature for 2 hours. The membranes were then washed with PBS Tween 20 and incubated with secondary antibody goat anti-mouse immunoglobulin G (IgG)/horseradish peroxidase (HRP) (1:1000, Beyotime) for 1 hour at room temperature. Enhanced chemiluminescence (ECL) detection of the target protein was performed using a Pierce ECL system (Thermo Scientific, Waltham, MA, USA). HSPA2 protein expression levels were quantified by Bio-Rad Image Lab software and presented as the densitometric ratio of the targeted protein to GAPDH.

### Immunohistochemistry

All specimens were fixed with 4% formaldehyde, dewaxed, embedded and cut into 4 μm serial sections. Briefly, antigen retrieval was carried out in 10 mmol/l citrate buffer (pH 6.0) for 15 minutes at 100°C in a microwave oven. Endogenous peroxidase activity was blocked with 3% hydrogen peroxide for 10 minutes at room temperature. The sections were then incubated overnight at 4°C with anti-HSPA2 antibody (Santa Cruz Biotechnology). After washing with PBS, sections were incubated with secondary antibodies for 30 minutes at 37°C. The sections were then washed three times with PBS and treated with 3,3’-diaminobenzedine (DAB) for approximately 5 minutes. Finally, the sections were counterstained with hematoxylin, dehydrated, mounted and examined by light microscopy. Negative controls were probed with PBS under the same experimental conditions.

Immunohistochemical staining was assessed by two independent experienced pathologists who were blinded to all clinicopathological features. Five high power fields in each specimen were randomly selected, in which nuclear and/or cytoplasmic staining was considered to be positive staining for HSPA2. A staining index (values 0 to 9), obtained as a product of staining intensity (0 to 3: 0 point, no intensity; 1 point, weak intensity; 2 points, moderate intensity; 3 points, strong intensity) multiplied by the proportion of immunopositive cells of interest (≤10% = 1; 10% to 50% = 2; ≥50% = 3). Tumors were categorized into three groups according to the final staining index: negative or weak staining (scored 0 to 3), moderate staining (scored 4 to 6) and strong staining (scored 7 to 9). ESCC patients were dichotomized into a negative expression group (negative and weak staining scored 0 to 3), and positive expression group (moderate and strong staining scored 4 to 9) in order to better analyze the prognosis between groups.

### Statistical analysis

All continuous variables were expressed as mean ± SD from at least three separate experiments. HSPA2 levels in ESCC samples, adjacent non-cancerous tissue and normal tissues were examined by Wilcoxon signed-rank test. The association between HSPA2 and clinicopathological features was analyzed using χ^2^ test. Survival curves were obtained using Kaplan-Meier curves and log-rank tests. Multivariate prognostic factors were examined by the Cox proportional hazards model. A value of *P* <0.05 was considered to be statistically significant. All statistical calculations were performed with SPSS Statistics 18.0 software (IBM, Armonk, NY, USA).

## Results

### Expression of HSPA2 in different esophageal tissues

**Figure 1:**
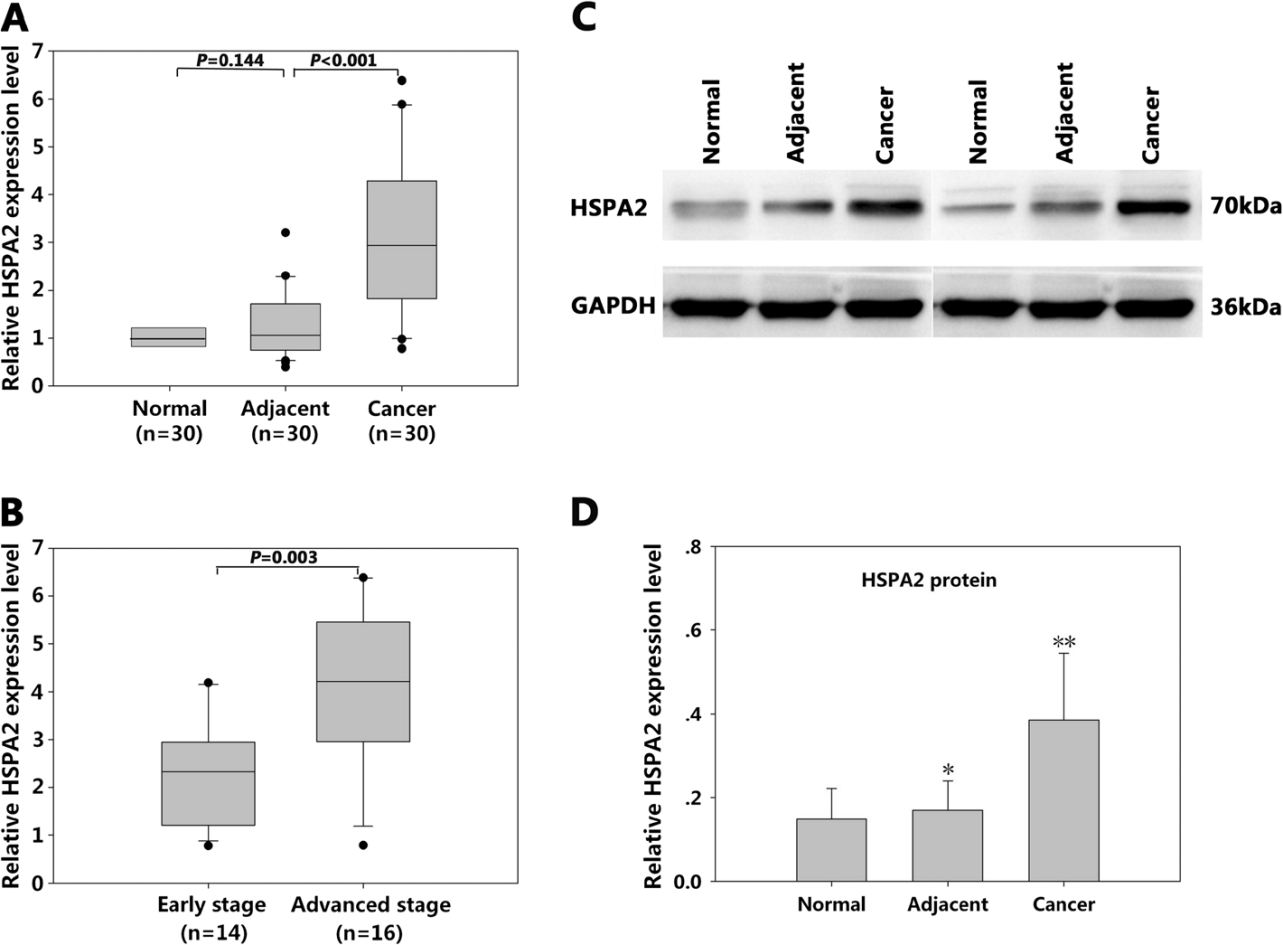
**Comparison of HSPA2 mRNA and protein levels in ESCC samples, adjacent non-cancerous tissues and normal tissues by qRT-PCR and western blotting.** (**A**) Relative HSPA2 mRNA expression levels in different tissues: cancer, 30 paired ESCC samples; adjacent, adjacent non-cancerous tissues; normal, normal esophageal tissues. (**B**) Relative HSPA2 mRNA expression levels in ESCC tissues at different stages: early and advanced. (**C**) Representative western blotting analysis of HSPA2 expression in all specimens. (**D**) Relative HSPA2 protein expression levels in different tissues. *P >0.05, compared with normal tissues; **P <0.05, compared with normal tissues and adjacent non-cancerous tissues. The relative HSPA2 protein expression level in ESCC tissues is about 2.67 times of that in normal esophageal tissues, and 2.35 times of that in adjacent non-cancerous tissues. Boxes represent the medians and interquartile ranges of the normalized threshold values. ESCC, esophageal squamous cell carcinoma; qRT-PCR, quantitative RT-PCR.

HSPA2 mRNA and protein expressions were detected in the 30 pairs of primary ESCC samples, adjacent non-cancerous tissues and normal tissues. The results in Figure 1A show that HSPA2 mRNA levels in the ESCC tissues were higher than in the adjacent non-cancerous tissues (3.1742 ± 1.6866 versus 1.2440 ± 0.6702, *P* <0.05). However, no significant differences were found between adjacent non-cancerous tissues and normal tissues (*P* >0.05). We also found that HSPA2 mRNA had different expression levels in the ESCC tissues at different tumor stages. As shown in Figure 1B, HSPA2 mRNA expression in advanced-stage ESCC tissues (n = 16) at stage III was significantly higher than in early-stage ESCC tissues (n = 14) at stages I and II (*P* <0.05). Western blotting analysis showed that the HSPA2 protein expression in ESCC tissues was significantly higher than in adjacent non-cancerous tissues and normal tissues (*P* <0.05) (Figure 1C, D).

### Correlation of HSPA2 expression with clinicopathological characteristics of ESCC tissues

**Figure 2:**
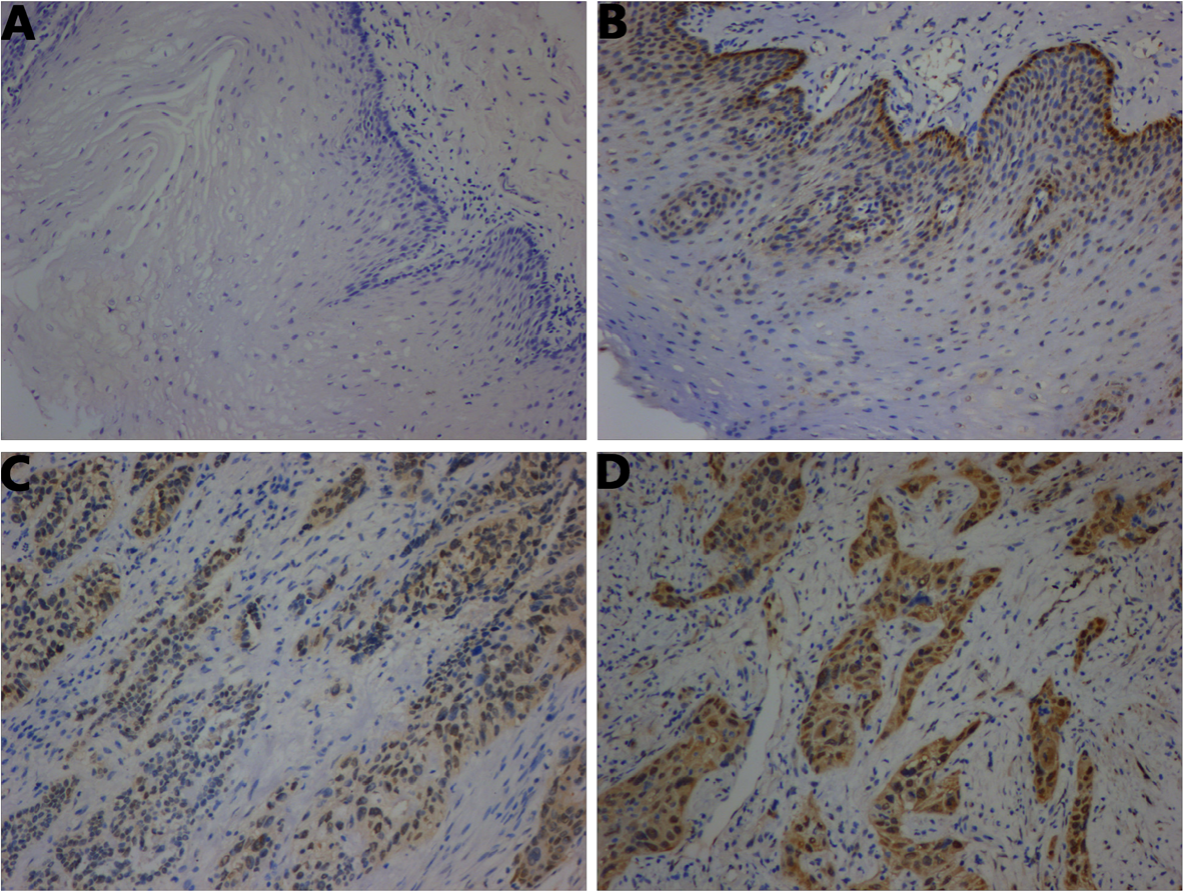
**Immunohistochemical analysis of HSPA2 in ESCC samples, adjacent non-cancerous tissues and normal esophageal tissues (original magnification × 100).** (**A**) Representative negative staining of HSPA2 in normal esophageal tissues. (**B**) Weak staining of HSPA2 in adjacent non-cancerous tissues. (**C**) Representative moderate staining and (**D**) strong staining of HSPA2 in ESCC tissues. The expression of HSPA2 was detected in cells of the basal layers of the esophagus (**B**), and HSPA2-positive staining was predominantly observed in the nucleus and some occurred in the cytoplasm in ESCC tissues (**C**,**D**). ESCC, esophageal squamous cell carcinoma.

A total of 120 pairs of paraffin-embedded ESCC and adjacent non-cancerous tissues were analyzed by immunohistochemistry. The positive expression rate of HSPA2 in ESCC tissues (90/120, 75%) was significantly higher than in the adjacent non-cancerous tissues (55/120, 45.83%, *P* <0.05). As shown in Figure 2, HSPA2-positive staining was predominantly observed in the nucleus and some occurred in the cytoplasm. HSPA2 are classified as cytosol/nuclear proteins able to translocate between the cytoplasm and nucleus [14]. At physiological temperature, the HSPA2 is localized primarily in cytoplasm, whereas the HSPA2 localization shifts to the nucleus and nucleoli in heat-hock cancer cells [14,23]. From immunohistochemical analysis, the clinicopathological data of the patients are summarized in Table. 1. Overexpression of HSPA2 was significantly associated with primary tumor, TNM stage, lymph node metastases and recurrence respectively (all, *P* <0.05). There were no significant differences in age, gender, drinking, tumor site or tumor grade.

### Prognostic significance of HSPA2 expression in ESCC patients

**Figure 3:**
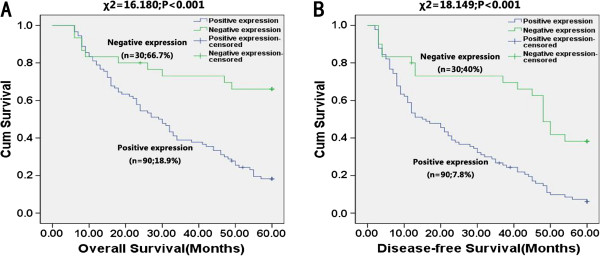
**Overall survival and disease-free survival curves constructed by Kaplan-Meier analysis, grouped by HSPA2 expression (negative expression, positive expression).** (**A**) Overall survival and (**B**) disease-free survival curves. Log-rank test (**A**: χ^2^ = 16.180, *P* <0.001; **B**: χ^2^ = 18.149, *P* <0.001) demonstrated that ESCC patients in the HSPA2-positive expression group had shorter overall survival and poorer disease-free survival compared with those in the HSPA2-negative expression group. Three patients were lost to follow-up because of telephone number changes or home moving. ESCC, esophageal squamous cell carcinoma.

At the end of clinical follow-up, survival information was available for 117 of 120 patients; three patients were lost to follow-up because of telephone number changes or home moving. Patients were divided into a HSPA2-positive group (n = 90) and a HSPA2-negative group (n = 30). Kaplan-Meier curves of survival analysis demonstrated that patients with HSPA2-positive expression had a shorter overall survival than patients with HSPA2-negative expression (survival rate: 18.9% versus 66.7%, *P* <0.001) (Figure 3). Analysis of disease-free survival also obtained a similar result (survival rate: 7.8% versus 40%, *P* <0.001). As shown in Table 2, univariate analysis showed that overall survival and disease-free survival were correlated with primary tumor, TNM stage, lymph node metastases and HSPA2 expression. Furthermore, Cox multivariate regression analysis indicated that HSPA2 expression, primary tumor and TNM stage were considered as independent prognostic factors for overall survival and disease-free survival in Table 3.

**Table 2:** Univariate survival analysis of overall survival and disease-free survival in 120 patients with ESCC

**Features**	**Overall survival**		**Disease-free survival**	
	**Hazard ratio (95% CI)**	***P*value**	**Hazard ratio (95% CI)**	***P*value**
Age (≤60/>60 years)	0.713 (0.456 to 1.116)	0.139	0.745 (0.493 to 1.125)	0.162
Gender (female/male)	0.915 (0.553 to 1.513)	0.728	0.975 (0.624 to 1.525)	0.913
Drinking (yes/no)	1.043 (0.670 to 1.622)	0.853	1.017 (0.681 to 1.518)	0.935
Tumor site (upper + lower/middle)	1.347 (0.797 to 2.277)	0.266	1.216 (0.768 to 1.926)	0.405
Grade (G2 + G3 + G4/G1)	1.137 (0.739 to 1.749)	0.559	1.070 (0.723 to 1.584)	0.735
Primary tumor (T3 + T4/T1 + T2)	2.157 (1.380 to 3.372)	0.001^a^	1.872 (1.256 to 2.791)	0.002^a^
TNM stage (III/I + II)	21.177 (11.057 to 40.561)	<0.001^a^	15.085 (8.320 to 27.350)	<0.001^a^
Lymph node metastases (−/+)	0.572 (0.369 to 0.887)	0.012^a^	0.625 (0.418 to 0.935)	0.022^a^
HSPA2 expression (positive/negative)	3.557 (1.827 to 6.922)	<0.001^a^	2.901 (1.721 to 4.888)	<0.001^a^

^a^*P* values considered statistically significant at <0.05. CI, confidence interval; ESCC, esophageal squamous cell carcinoma; G, grade; N, lymph node metastases; T, primary tumor**.** Drinking alcohol is defined as: average amount of drinking alcohol is 50 grams or more per day, and period of drinking continues to 1 year or more. Drinking alcohol parameter: amount of drinking/ day **×** years of drinking.

**Table 3:** Multivariate survival analysis of overall survival and disease-free survival in 120 patients with ESCC

**Features**	**Overall survival**		**Disease-free survival**	
	**Hazard ratio (95% CI)**	***P*value**	**Hazard ratio (95% CI)**	***P*value**
Primary tumor (T3 + T4/T1 + T2)	1.746 (1.104 to 2.762)	0.017^a^	1.516 (1.005 to 2.289)	0.048^a^
TNM stage (III/I + II)	16.347 (8.560 to 31.215)	<0.001^a^	11.729 (6.448 to 21.335)	<0.001^a^
Lymph node metastases (−/+)	0.838 (0.534 to 1.315)	0.442	0.906 (0.596 to 1.375)	0.642
HSPA2 expression (positive/negative)	2.210 (1.101 to 4.435)	0.026^a^	2.115 (1.222 to 3.661)	0.007^a^

^a^*P* values considered statistically significant at <0.05. CI, confidence interval; ESCC, esophageal squamous cell carcinoma; G, grade; N, lymph node metastases; T, primary tumor.

## Discussion

The *HSPA2* gene was originally characterized as a counterpart of the testis-specific rodent *HST70*/*HSP70-2* gene, which was specially and highly expressed in the testis [5,6]. Recently, research has shown that human tumor cells and some somatic tissues can express this gene at significant levels [7-10,12-14]. The polymorphism of HSPA2 at position 1267 has been suggested to be associated with carcinogenesis in many malignant cancer tissues [7-10]. The highest level of HSPA2 is also detected in cells of the basal layers of the esophagus [23], but the function of HSPA2 in basal cells, which may be involved in the proliferation and/or differentiation of epidermal keratinocytes, is currently unknown.

In this study, the expression levels of HSPA2 mRNA and protein in the ESCC tissues were significantly higher than in adjacent non-cancerous tissues and normal esophageal tissues, consistent with other cancer reports [7-9]. As a chaperone protein, HSPA2 is essential for the growth of spermatocytes and cancer cells, and it is known to be involved in apoptosis and regulation of cell proliferation [18]. Silencing the expression of the *HSPA2* gene by RNA interference suggests that HSPA2 increases the growth rate and tumorigenic potential not only in the cell culture but also in tumor xenograft in mice [7-9,15]. However, the underlying mechanisms for the high expression of HSPA2 in tumors remain incompletely understood, but likely involve regulatory processes, such as cell cycling. Indeed, HSPA2 appears to be a molecular chaperone for CDC2 and is required for CDC2/cyclinB1 complex formation, whose destruction can prevent development of the CDC2 kinase activity, required to trigger G2/M phase transition [24]. During meiosis, spermatogenic cells synthesize HSPA2, knockout of HSPA2 in HSPA2 (−/−) male mice testes induce primary spermatocytes to arrest in meiosis I and undergo a dramatic increase in spermatocyte apoptosis, which was leading to male infertility [6]. HSPA2 overexpression protects V79 fibroblasts against bortezomib-induced apoptosis [25]. The mechanism of a mutated HSPA2 chaperone has a dominant effect in tumor cells by triggering the G2/M phase transition during the mitotic cell cycle, which could explain the HSPA2 abnormal expression in somatic tumors. Moreover, downregulation of lens epithelium-derived growth factor (LEDGF) is responsible for the death of HSPA2-depleted cancer cells and LEDGF possesses great oncogenic potential [26]. HSPA2 may also have a potential role in cancer pathogenesis by participating in the regulation of antitumor immunity, such as acting as a chaperone molecule for immunogenic tumor-associated peptides [27], while HSPA2 has been identified as a putative susceptibility locus in organ-specific autoimmune diseases. Lastly, a new regulatory mechanism of HSPA2 expression in tumor cells has been disclosed, which suggests that the upregulation of HSPA2 enhanced the resistance of tumor cells to hypoxia-induced apoptosis, which provides a new insight into how tumor cells overcome hypoxic stress and survive [28].

Immunohistochemical analysis of a large set of specimens revealed that HSPA2 protein was positively expressed in 75% (90/120) ESCC tissues, compared to 45.83% (55/120) in adjacent non-cancerous tissues (*P* <0.05). We correlated HSPA2 protein expression with various clinicopathological factors in 120 ESCC patients. Our results showed that overexpression of HSPA2 was significantly associated with primary tumor, TNM stage, lymph node metastases and recurrence respectively (all, *P* <0.05). We also found HSPA2 mRNA expression in advanced-stage ESCC tissues at stage III was significantly higher than in early-stage ESCC tissues at stages I and II. These data revealed a potential association between HSPA2 overexpression and malignant phenotypes, such as metastasis, consistent with other reports in breast cancer [7], cervical cancer [8] and bladder urothelial carcinoma [9]. It is well known that the HSPA2 is localized primarily in cytoplasm at physiological temperature, whereas HSPA2 migrates to the nucleus and nucleoli under heat-shock conditions [14,23], and our results confirm this theory. The phenomenon of HSPA2 translocation into the nucleus and nucleoli has been presently found in many other heat-shock cancer cells, which is part of a cellular protective response under stress conditions, but the mechanism is currently unknown. Generally, HSPA2 is involved in intracellular trafficking and nuclear receptor binding, which also prevents inappropriate protein aggregation and mediates transport of immature (or damaged) proteins to target subcellular compartments for final packaging, degradation or repair [29]. Since HSPA2 shuttles continuously between the cytoplasm and nucleus during heat-shock, this cell migration is critical for tumor formation and metastasis.

It is widely accepted that metastasis contributes to the high mortality rate for patients with ESCC. Our data revealed that high HSPA2 protein expression was well correlated with lymph node metastasis and predicted worse prognosis. Although close association between HSPA2 expression and ESCC metastasis has been established in our study, the possible mechanisms are still unclear and need further investigation. Moreover, lymph node metastasis is one of the vital elements of TNM stage. It is also well known that lymph node metastasis frequently occurs in advanced-stage ESCC patients. Therefore, it is not difficult to explain the reason for overexpression of the *HSPA2* gene in late stages. The abnormal excessive proliferation of tumor cells also frequently occurs in late stages and overexpression of HSPA2 can promote development of the CDC2 kinase activity, required to trigger G2/M phase transition and regulate cell proliferation through controlling cell cycling. The overexpression of HSPA2 leads to the prolonged cell cycling arrest at the G2 phase. Most importantly, the resistance of tumor cells to hypoxia-induced apoptosis in late stages is very strong and the new regulatory mechanism mentioned above indicates that the upregulation of HSPA2 may enhance the resistance of tumor cells to hypoxia-induced apoptosis. All these theories illustrate that overexpression of HSPA2 promotes the occurrence and development of ESCC.

Finally, patients with HSPA2-positive expression were significantly associated with shorter disease-free and overall survival by using the Kaplan-Meier analysis. Therefore, HSPA2 protein expression pattern might be a valuable prognostic marker for ESCC patients. More importantly, from univariate and multivariate analysis, we obtained sufficient evidence to deduce that HSPA2 expression level was an independent prognostic indicator for patients with ESCC. Overexpression of HSPA2 is correlated with poor therapeutic outcomes in human breast cancer, cervical cancer and bladder urothelial cancer; furthermore, it is now a valuable prognostic marker for breast cancer patients [7]. The polymorphism of HSPA2 acts as an attractive susceptibility marker and independent prognostic indicator in Kangri cancer patients [30]. Considering the frequency of positivity of HSPA2 in adjacent non-cancerous tissues is much higher, whether or not HSPA2 molecule can be qualified to be a prognostic biomarker will need to be validated in future clinical work.

## Conclusions

Our present study demonstrated that elevated HSPA2 expression levels was positively associated with the progression and poor prognosis in patients with ESCC, which indicated that HSPA2 might serve as a valuable prognosis marker for ESCC patients. However, the possible underlying mechanisms for its participation in ESCC progression are still unclear. Therefore, the next step we will take will be further research of the cell signaling pathway in order to gain a better molecular mechanism understanding in this field.

## Abbreviations

CI: Confidence interval; CTL: Cytotoxic T lymphocyte; DAB: 3,3’-diaminobenzedine; ECL: Enhanced chemiluminescent; ESCC: Esophageal squamous cell carcinoma; GAPDH: Glyceraldehyde 3-phosphate dehydrogenase; HRP: Horseradish peroxidase; HSPA2: Heat shock-related 70 kDa protein 2; IgG: Immunoglobulin G; LEDGF: Lens epithelium-derived growth factor; PBS: Phosphate buffered saline; qRT-PCR: Quantitative RT-PCR; RT-PCR: Reverse transcription polymerase chain reaction; SLE: Systemic lupus erythematosus.

## Competing interests

The authors declare that they have no competing interests.

## Authors’ contributions

ZH carried out the qRT-PCR and western blotting, participated in the sequence alignment and drafted the manuscript. CW carried out the immunohistochemistry. DCJ participated in the design of the study and performed the statistical analysis. ZCF conceived of the study, participated in its design and coordination, and helped to draft the manuscript. All authors read and approved the final manuscript.
